# High-Resolution Hyperspectral Imaging Using Low-Cost Components: Application within Environmental Monitoring Scenarios

**DOI:** 10.3390/s22124652

**Published:** 2022-06-20

**Authors:** Mary B. Stuart, Matthew Davies, Matthew J. Hobbs, Tom D. Pering, Andrew J. S. McGonigle, Jon R. Willmott

**Affiliations:** 1 Department of Electronic and Electrical Engineering, University of Sheffield, Sheffield S1 4DE, UK; mbstuart1@sheffield.ac.uk (M.B.S.); matt.davies@sheffield.ac.uk (M.D.); m.hobbs@sheffield.ac.uk (M.J.H.); 2 Department of Geography, University of Sheffield, Sheffield S10 2TN, UK; t.pering@sheffield.ac.uk (T.D.P.); a.mcgonigle@sheffield.ac.uk (A.J.S.M.)

**Keywords:** hyperspectral, low-cost, high-resolution, environmental monitoring

## Abstract

High-resolution hyperspectral imaging is becoming indispensable, enabling the precise detection of spectral variations across complex, spatially intricate targets. However, despite these significant benefits, currently available high-resolution set-ups are typically prohibitively expensive, significantly limiting their user base and accessibility. These limitations can have wider implications, limiting data collection opportunities, and therefore our knowledge, across a wide range of environments. In this article we introduce a low-cost alternative to the currently available instrumentation. This instrument provides hyperspectral datasets capable of resolving spectral variations in mm-scale targets, that cannot typically be resolved with many existing low-cost hyperspectral imaging alternatives. Instrument metrology is provided, and its efficacy is demonstrated within a mineralogy-based environmental monitoring application highlighting it as a valuable addition to the field of low-cost hyperspectral imaging.

## 1. Introduction

High spatial and spectral resolution hyperspectral imaging is becoming increasingly important for a wide range of industries. It has reached a reasonable level of maturity in agriculture. It promises to be a beneficial measurement modality that can provide datasets capable of resolving intricate details and variations across a broad range of targets. Continued uptake of the technology will require a reduction in cost of hardware along with an increased knowledge of the meaning behind the spectra for any particular application. Despite the benefits, high-spatial-resolution hyperspectral imaging can be difficult to achieve due to the associated trade-offs between spatial resolution, spectral resolution, and signal-to-noise ratio [1,2]. These factors represent key performance parameters within instrumentation design, having a significant impact on the overall abilities of the final configuration [3,4,5]. The instrumentation design process, therefore, often becomes a balancing act, finding the best possible combination of these three factors that allows the highest quality data to be captured from the chosen application. Whilst these trade-offs affect hyperspectral imaging applications as a whole, many low-cost designs are typically more adversely affected as a result of their use of lower cost components. This results in many low-cost hyperspectral instruments foregoing high spatial resolution in order to achieve effective spectral outputs, and, in turn, this limits high-spatial-resolution hyperspectral imaging to more costly commercial instrumentation [6,7]. These limitations have further repercussions; by placing these high-resolution datasets ‘out-of-reach’ for many less-well-resourced research teams and organisations, it hinders the continued democratisation of hyperspectral imaging techniques and, in so doing, limits a wide range of data collection applications. Low-cost high-resolution hyperspectral imaging, therefore, represents a critical area for continued development. By developing instrumentation that is capable of accurate spectral identification of small-scale spatial targets, it enables a broad range of more detailed spectral measurements that can provide key knowledge and understanding in a variety of application areas. Low-cost hyperspectral imaging will not only open up the technology to a wider range of applications, but it will also enable an increase in knowledge of the correlations/causations between spectra and the parameters that can be used to improve, e.g., manufacturing processes, by hugely expanding the user base.

In environmental monitoring applications, high-spatial-resolution hyperspectral imaging can enable the capture of intricate features that would often be overlooked by traditional monitoring methods. This approach is, therefore, employed across a wide variety of applications, from spatially complex environments such as swamps [8], or dense forest canopies [1], to the accurate identification of volcanic gases [3,9]. Many existing applications focus on natural and agricultural vegetation monitoring [10,11,12,13,14], however, there has also been a recent increase in interest in high-resolution analyses for mining operations [15,16,17], geological exploration [18,19,20], mineralogy [21,22,23,24,25] and petrology [23,26,27,28,29]. Hyperspectral imaging provides a rapid, non-destructive, and information-rich means of data collection [15,23,30], enabling both an increase in our understanding of the structure and composition of key environmental settings whilst also providing valuable planetary analogues for continued solar system exploration [30,31,32,33,34,35]. The application of high-spatial-resolution hyperspectral imagers within these scenarios is, therefore, of considerable benefit.

To date, whilst high-spatial-resolution instrumentation has become more common place within these applications, there remains a considerable gap in the existing literature surrounding the application of low-cost instrumentation within these domains. Whilst many existing low-cost alternatives are capable of accurate and detailed data capture, making them valuable additions to the research field, very intricate targets, ca. < 1 mm, are often not easily resolved by these approaches. In this article we, therefore, introduce a Low-Cost High-Resolution hyperspectral imager as an accessible alternative to existing measurement and monitoring approaches. Within this article we use “high-resolution” to refer to data capture quality required to be associated with high definition (HD) video, however, given the final processed output datasets are not a video format, we have chosen not to use HD more broadly within the text. Furthermore, “high-resolution” can also be attributed to the spectral resolution of this instrument. With a spectral resolution of 0.29 nm, this instrument compares favorably with a broad range of existing instrumentation [6]. Additionally, we use the term “low-cost” to refer to instrumentation that is significantly cheaper than the typical cost of commercially available systems. Commercial hyperspectral imaging instrumentation often cost more than £30,000, with some systems costing up to £150,000 [7]. In comparison, the instrument detailed within this article costs ca. £11,000 to develop, with the majority of these costs associated with the chosen camera sensor. This single-instrument cost would fall significantly if our design were to be supplied commercially due to the inverse relationship between sales volume and price. The instrument is semi-portable and capable of mm-scale spatial data acquisitions. In this article we aim to present a thorough analysis of, and introduction to, our Low-Cost High-Resolution hyperspectral imager, providing insights that demonstrate its significant potential. Instrument design and metrology are presented before its application within a mineralogy-based study with the aim of demonstrating the instrument’s efficacy and potential within environmental monitoring contexts. In so doing, we highlight the significant potential offered by this Low-Cost High-Resolution instrument, demonstrating it to be a valuable addition to the research field and an additional step towards the wide-spread democratisation of hyperspectral imaging techniques.

## 2. Materials and Methods

**Figure 1:**
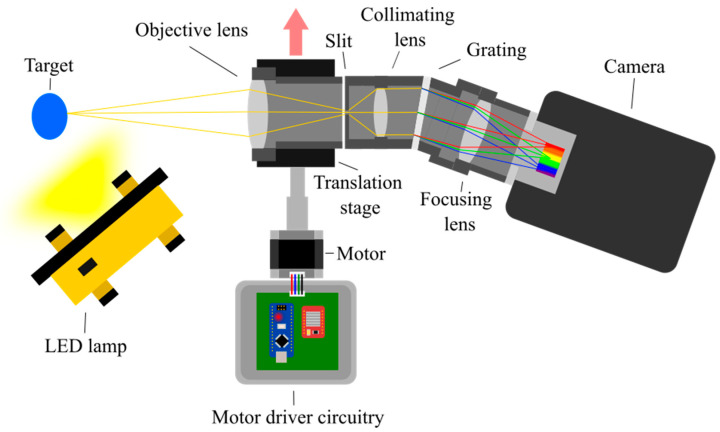
Schematic diagram of the Low-Cost High-Resolution hyperspectral imager showing how axial and marginal rays pass through the optical system. Blue, green, and red lines represent example wavelength rays after diffraction has taken place. Not to scale.

**Figure 2:**
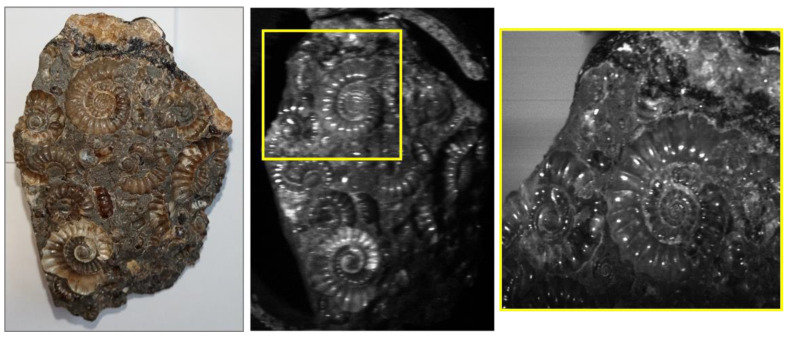
Example frames of an ammonite fossil taken from a hyperspectral data cube demonstrating the spatial resolution possible with this instrument. The first panel shows a standard color image of the target for reference. The additional panels show hyperspectral frames captured at focal lengths of 18 mm and 55 mm, respectively.

The Low-Cost High-Resolution hyperspectral imager (Figure 1) is a semi-portable instrument, capable of capturing spectral information from mm-scale spatial targets, and focus can be adjusted to best fit the intended target. This is demonstrated within Figure 2, which shows an example of the data quality capture possible with this set-up using two different focal lengths. The instrument is composed of commercially available components, as listed in Table 1. A key benefit of the Low-Cost High-Resolution instrument is its inherent modularity. This enables key components and their configuration to be altered to best fit the intended application without compromising the overall abilities of the imager. The components listed in Table 1 were selected to best fit the intended applications discussed within this article, however, many of them can be altered or exchanged, enabling a wider range of applications with the additional benefit of potential cost reduction if required. For example, the width of the slit can be adjusted without disturbing the existing set-up, enabling the capture of a greater range of target scenes under variable illumination conditions. Furthermore, for targets with key spectral features outside of the existing range of this instrument, the diffraction grating can be replaced with an alternative with relative ease. Finally, the Hamamatsu C13440 camera (Hamamatsu, Shizuoka, Japan) makes up a considerable portion of the development costs for this particular design. It could be replaced with a lower cost alternative, for example, a Thorlabs Quantalux CS2100M-USB (Thorlabs, Newton, NJ, USA). Of course, replacing the camera with a considerably lower cost alternative, will influence the data capture quality. However, the operator can look to determine the acceptable limitations and trade-offs between data quality and cost reduction within the specifics of their intended application. The ease of these alterations highlight the versatility of this approach to instrument design, demonstrating the significant potential for versatile, low-cost, high-resolution hyperspectral instrument development for a range of applications and research fields, including potential adaptations for the capture of longer wavelengths within the infrared, however, these alterations would likely result in a significant increase in development costs.

**Figure 3:**
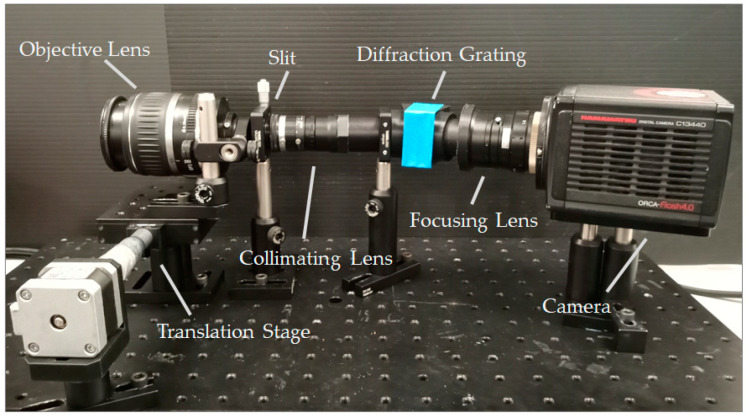
The Low-Cost High-Resolution hyperspectral imager within a laboratory setting.

In its current form (Figure 3), the instrument is capable of detecting spectral information across the visible spectrum (450–650 nm), however, given its inherent modularity this wavelength range can be altered with relative ease. The wavelength range of the instrument is limited by the focusing lens, which produced mild vignetting. We, therefore, chose to partially crop the sensor, sacrificing some of the spectral range. This could be avoided by replacing the focusing lens or selecting a diffraction grating with a lower groove density. However, it should be noted that whilst replacing the diffraction grating would enable a greater spectral range to be captured, it would result in a trade-off, reducing the spectral resolution. The instrument is semi-portable. By referring to the instrument as “semi-portable” we intend to highlight its increased maneuverability over traditional laboratory-based hyperspectral imagers. This instrument can be operated using a laptop, removing its reliance on a static computer terminal. This enables it to be utilised in a wider range of data collection scenarios, increasing its range of potential applications.

**Figure 4:**
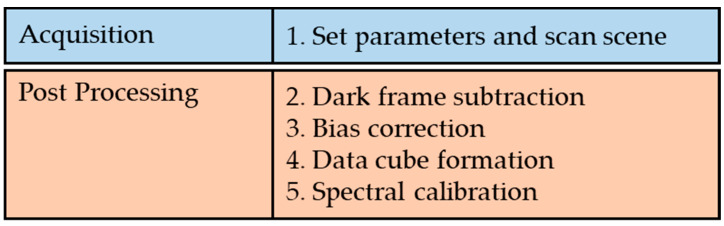
Workflow used to capture a hyperspectral image with the Low-Cost High-Resolution instrument detailing image acquisition and post processing stages.

To obtain a hyperspectral image with this instrument, the objective lens is translated across the scene using a compact translation stage travelling at a rate of 0.2 mm/s providing stable and reliable scene capture that is unaffected by factors such as operator shake. Using this method, a full hyperspectral scene could be captured in 1 min. The scanning range is determined by the fore optics, and, as such, can be altered/replaced to better fit a range of larger and smaller targets e.g., using scanning mirrors or microscope coupling respectively. Illumination under laboratory conditions is provided by a 20 W LED lamp. The instrument is controlled using HC Image Live (version 4.3.1.30, Hamamatsu) software. The software can be used to tailor the camera settings to the specifics of the chosen application; factors such as exposure time, and image dimensions can be altered by the operator. Similarly, the focal length, and working distance can be altered to best fit the chosen scene. The settings utilised for the data capture discussed within this article are shown in Table 2. After data capture was completed the hyperspectral data cube was built within MATLAB to create a visual representation of the acquired dataset. Spectral datasets were also corrected for sensor and illumination biases within this software allowing the true spectral response curves of each target scene to be extracted for further examination and analysis. To do this, white and dark references were obtained during the image capture phase. Note the white reference utilised was a piece of matt white card illuminated in the same manner as the target scene. Figure 4 shows the workflow required to capture a hyperspectral image, highlighting the sequence of steps as they were implemented.

**Figure 5:**
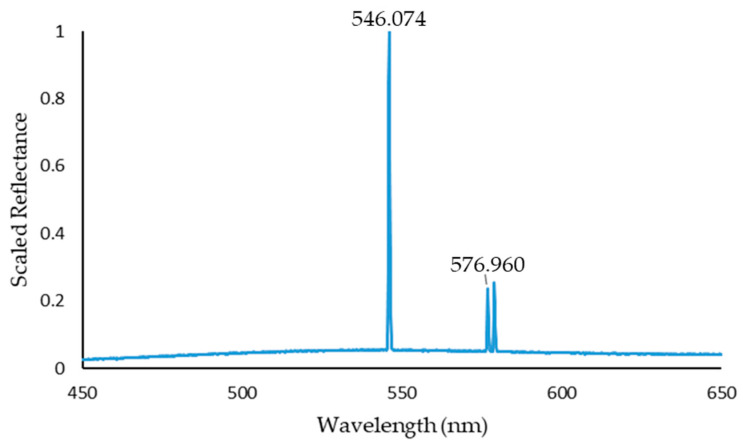
Spectrum captured from a Mercury Argon lamp using the Low-Cost High-Resolution instrument highlighting the peaks present at 546.074 nm and 576.960 nm that were used to spectrally calibrate the instrument.

Spectral calibration was completed using a Mercury Argon lamp that produced a series of intense narrow peaks at known wavelengths. In this research we utilised the peaks present at 546.074 nm and 576.960 nm, as shown in Figure 5. These two known points were used to calculate the wavelength range and the increment present between each value. The full width at half maximum (FWHM) was calculated using the 546.074 nm peak and was found to be two pixels.

The Instantaneous Field of View (IFOV) for each pixel within the spectrometer image was measured to be approximately 2 mm × 2 mm (18 mm focal length) and 300 μm × 300 μm (55 mm focal length) for a 95% energy enclosure at a working distance of 300 mm. The Total Field of View (TFOV) is determined by the slit height relative to the image circle of the objective lens and the travel distance of the translation stage. To calibrate the instrument, allowing samples from different sources to be accurately compared, a radiometric assessment of the optical power represented by each pixel within the image was performed by measuring the power reflected by the white reference target. This was performed using a photodiode-based radiometer, described by Zhu et al. [36]. The RG850 long-pass filter was replaced by a Thorlabs narrow bandpass filter (#FB550-10), centered on 550 nm with a FWHM of 10 nm. The reflected optical power collected by the radiometer was calculated by comparing the photocurrent measured by the radiometer with and without the filter in the optical path. Given that the FOV of the radiometer represented an area upon the target of approximately 14 mm in diameter at its 1 m operating distance, the reflected power per unit area, without the filter in place, was calculated from this to be approximately 250.26 μW/m^2^. The optical power reflected from the illumination reference target was, therefore, estimated to be 38.51 nW.

## 3. Results

### Optical Characterisation

**Figure 6:**
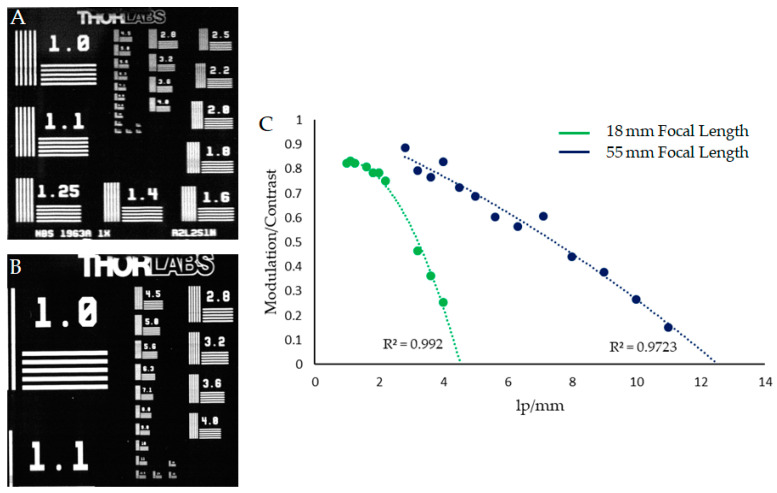
CTF analysis for both focal lengths. (**A**,**B**) (**left**) show an image frame of the resolution target captured at an 18 mm focal length and a 55 mm focal length, respectively, (**C**) (**right**) shows the resulting CTF values for horizontal line pairs.

**Figure 7:**
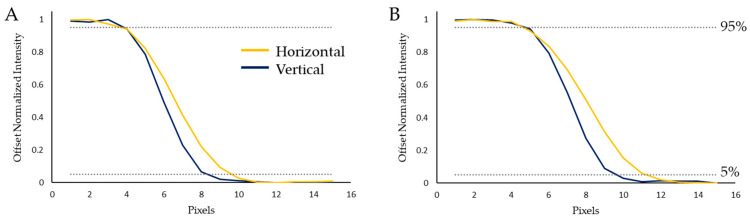
Knife-edge measurements for each focal length. (**A**) shows the results for the 18 mm focal length demonstrating a one-pixel discrepancy between orientations. (**B**) shows results for the 55 mm focal length demonstrating a two-pixel discrepancy between orientations.

To provide a quantifiable measure of the optical abilities of the instrument, the Contrast Transfer Function (CTF) was calculated for both focal lengths used within this article. The modulation depth was measured with a Thorlabs R2L2S1N resolution target. Images were captured of the target and the modulation depth for a number of line pair widths was calculated (Figure 6). The modulation depth was different for horizontally and vertically orientated CTF targets. This is due to the scanning nature of the system. The optical resolution was measured from the horizontal CTF targets (as shown in Figure 6) because the vertical CTF targets were influenced by small variations in scan speed, minor perturbations in the translation stage, and the finite slit width. A knife-edge measurement provides a simple method of determining the point-spread-function of an optical system [37]. The point-spread-function quantifies the extent to which an optical system can resolve a point source of light. A knife-edge was, therefore, used to assess the influence of these sources of error on the resolution. The width in pixels between 5% and 95% of the measured signal normal to the knife-edge was measured vertically (parallel to the slit) and horizontally (normal to the slit). Figure 7 shows the horizontal and vertical knife-edge measurements for both focal lengths. In this figure it is clear that the vertical knife-edge measurements are better than the horizontal measurements, as expected. However, given the discrepancy of one pixel for the 18 mm focal length, and two pixels for the 55 mm focal length, this difference does not appear to be great enough to significantly influence the quality of output datasets acquired with this instrument.

## 4. Discussion

### Example Application

To demonstrate both the spectral and spatial abilities of the Low-Cost High-Resolution hyperspectral imager within an environmental monitoring-based application, we chose to focus within the field of mineralogy. In Section 1 we highlighted the importance of resolving highly detailed spectral and spatial datasets within this discipline, therefore, through the following measurements we aim to highlight the efficacy of our instrumentation within this important area of research. Furthermore, the existing literature highlights the general absence of low-cost hyperspectral imaging applications within this domain, therefore, we aim to provide a foundation for further developments in low-cost hyperspectral imaging techniques within this field.

**Figure 8:**
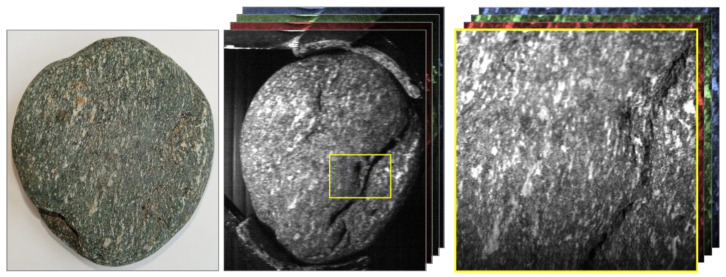
Hyperspectral image frames of a gneiss sample demonstrating the spatial resolution of this instrument. Characteristic banding and surface features are clearly visible within the hyperspectral data and can be easily related to their specific location on the original target. The image on the left is a standard color image of the sample and the hyperspectral images are on the right-hand side of the figure. The hyperspectral images are just one slice through the data cube that contains 689 discrete wavelength values. RGB frames represent the availability of different wavelength frames within the hyperspectral data cube.

A variety of rock samples exhibiting intricate crystal structures and surface variations were imaged to demonstrate the clarity of datasets the instrumentation was capable of capturing. Figure 8 shows a gneiss sample with characteristic banding. Looking at the hyperspectral frames of this sample, the quality of the spatial data resolution is clearly demonstrated. The sample can be clearly identified within the hyperspectral data, and exact locations can be determined for further, more detailed analysis if required. This is of significant benefit within the field of mineralogy, enabling the precise spectral response of specific sample locations to be observed and monitored effectively.

**Figure 9:**
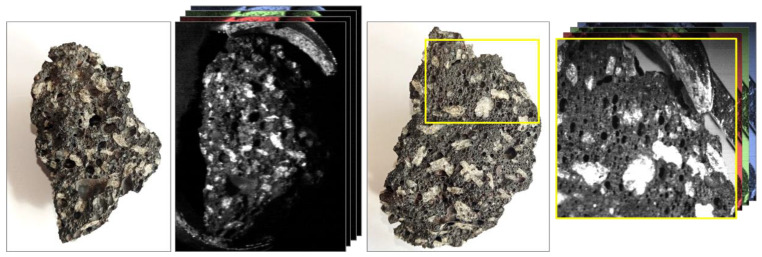
Two hyperspectral image frames of a basalt sample compared to standard color images. Note the clarity of the surface features within the hyperspectral frames allowing clear differentiation between feldspar and surface features. The hyperspectral images are just one slice through the data cube that contains 689 discrete wavelength values. RGB frames represent the availability of different wavelength frames within the hyperspectral data cube.

Similarly, when presented with a more complex target, the instrument was shown to perform well. Figure 9 demonstrates this using a basalt sample with plagioclase feldspars. The figure highlights this sample has a greater surface complexity with irregular surface variations, and bubble structures present alongside the feldspar features. This provides a much greater challenge for effective hyperspectral image collection; however, this figure demonstrates that the Low-Cost High-Resolution instrument is capable of accurately detecting these irregular features, clearly identifying individual mm-scale targets.

**Figure 10:**
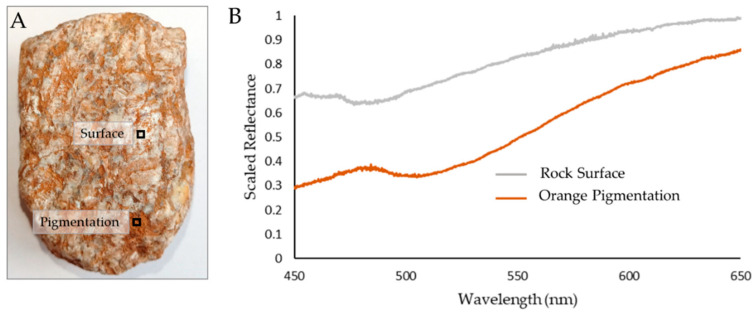
Spectral data for a piece of supraglacial debris with orange pigmentation. (**A**) shows a standard color image of the rock sample highlighting the approximate locations that correspond to the spectral curves shown in (**B**).

It should be noted that the hyperspectral image frames shown within the figures of this article only represent single slices of a data cube spanning 689 discrete wavelength values. This means that for each imaged scene the instrument builds a 689 Mega Pixel (MP) image (1000 × 1000 × 689). Each image frame, therefore, represents a small piece of the total data available. This is demonstrated in Figure 10 which shows the spectral graphs that demonstrate the wealth of underlying data. Subtle changes in spectral response can be accurately attributed to specific locations by effectively visualizing these small-scale features within the hyperspectral images. Figure 10 shows a sample of glacial debris with clear variations across its surface. These variations can be clearly identified within the graphed spectral responses. Furthermore, the spectral response recorded across this target correlates well with expectation; areas of the rock surface display a generally brighter response across the visible spectrum, whilst areas with orange pigmentation display more limited reflectance across shorter wavelengths.

**Figure 11:**
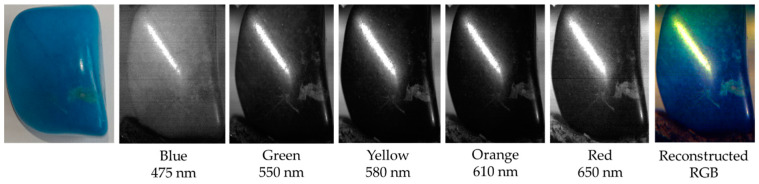
Spectral and spatial information obtained for a sample of lapis lazuli. Note the expected increase in reflectance across blue wavelengths followed by a steady reduction in reflectance towards longer wavelengths. The hyperspectral images represent single slices through the data cube that contains 689 discrete wavelength values. The reconstructed RGB image is created using red-green-blue equivalent images taken from the hyperspectral data cube.

**Figure 12:**
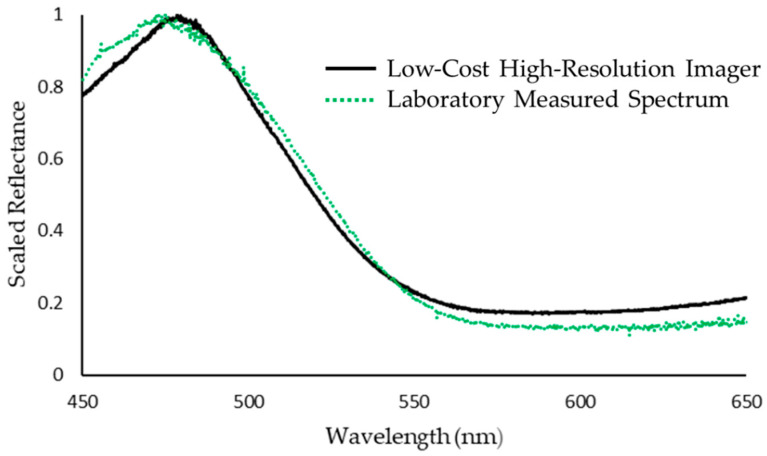
Spectral data obtained from a sample of lapis lazuli. Deviations from the laboratory-measured spectrum are associated with regions of low signal within the illumination spectrum. Note the correlation between the spectral response curve and the spectral-spatial data shown in Figure 11.

This is further demonstrated in Figure 11, which shows the spectral and spatial information captured for a sample of lapis lazuli. The hyperspectral data clearly shows a distinct peak in reflectance across blue wavelengths followed by a steady decline in reflectance towards red wavelengths, with areas of lighter, near-white, surface pigment becoming more obvious, across these generally darker wavelengths. This response is to be expected given the distinct visual coloring of the sample, and correlates well with the spectral response graph (Figure 12), which, in turn, matches with the known spectral response of this target [38].

The data discussed above clearly demonstrates the high spatial and spectral resolution achievable with the Low-Cost High-Resolution hyperspectral imager. The accurate identification of specific areas of spectral change is of significant benefit to a broad range of environmental monitoring applications and beyond. By clearly highlighting areas of specific spectral change, it can enable targeted analysis and further investigation. This, in turn, enables the thorough analysis of intended targets with minimal disruption and/or invasive analysis. Within the field of mineralogy these benefits can be particularly pertinent, increasing the accuracy of target studies whilst also minimizing the need for invasive investigation. Furthermore, these benefits remain in high demand across a broad range of applications, particularly within a low-cost, more accessible alternative. There is, therefore, significant potential to expand the use of low-cost high-resolution hyperspectral instrumentation across a variety of fields and disciplines. The datasets discussed within this article have shown the Low-Cost High-Resolution instrument to be capable of highly detailed data capture and analysis, demonstrating it to be a valuable addition to the research field.

The ability to capture high-resolution hyperspectral datasets using low-cost components outside of a laboratory setting is highly sought-after, enabling non-invasive in situ analyses, and removing the need for sample collection and preparation. This would be of significant benefit to a broad range of applications, particularly where vulnerable and/or fragile environmental settings are the focus of the intended study. The Low-Cost High-Resolution hyperspectral imager provides an opportunity to continue to improve the availability and accessibility of hyperspectral imaging techniques, adding to a range of low-cost alternative devices suitable for a wide variety of situations and application areas. By demonstrating the abilities of the Low-Cost High-Resolution hyperspectral imager, we have provided a further step towards this realisation, demonstrating the significant potential within the continued development of low-cost hyperspectral imaging alternatives.

## 5. Conclusions

In this article we have successfully demonstrated a low-cost, high-resolution hyperspectral imager capable of resolving mm-scale spatial targets. The instrument can produce a 689 MP image of a chosen scene, with a 50% modulation of 3 lp/mm and 7.1 lp/mm for focal lengths of 18 mm and 55 mm respectively. The efficacy of this imager was demonstrated within the field of mineralogy, clearly emphasising its spectral and spatial abilities, as well as demonstrating its proficiency within, and value to, the field of low-cost hyperspectral imaging in environmental monitoring. The instrument was shown to be capable of resolving a range of mm-scale targets across a variety of samples with different surface features and complexities. The accurate identification of these features within the hyperspectral data provides substantial benefits, significantly increasing the quality and accuracy of the acquired hyperspectral datasets without the expected costs. The Low-Cost High-Resolution hyperspectral imager provides a solid foundation for further innovations into the development of accessible high-resolution hyperspectral imaging, and in turn provides a valuable step towards the democratisation of hyperspectral imaging techniques as a whole.

## Figures and Tables

**Table 1:** Components of the Low-Cost High-Resolution hyperspectral imager.

Component	Part Used
Objective Lens	Canon EF-S 18–55 mm
Slit	Thorlabs VA100C (set at 300 μm).
Collimating Lens	Thorlabs MVL75M1 75 mm telephoto c mount
Transmission Diffraction Grating	Edmund Optics #49-580
Focusing Lens	Thorlabs MVL50M23 50 mm telephoto c mount
Camera Sensor	Hamamatsu C13440

**Table 2:** Data capture settings used for the High-Resolution hyperspectral imager.

	Setting
Exposure Time (ms)	60
Wavelength Range (nm)	450–650
Spectral Resolution (FWHM) (nm)	0.29
Spatial Resolution (pixels)	1000 × 1000
Focal Lengths (mm)	18 and 55

## Data Availability

All relevant data are shown in the paper or could be recreated by following the methodology in the paper.
